# *MfPIF1* of Resurrection Plant *Myrothamnus flabellifolia* Plays a Positive Regulatory Role in Responding to Drought and Salinity Stresses in *Arabidopsis*

**DOI:** 10.3390/ijms21083011

**Published:** 2020-04-24

**Authors:** Jia-Rui Qiu, Xiang-Ying Xiang, Jia-Tong Wang, Wen-Xin Xu, Jia Chen, Yao Xiao, Cai-Zhong Jiang, Zhuo Huang

**Affiliations:** 1 College of Landscape Architecture, Sichuan Agricultural University, Wenjiang 611130, China; qiujiarui@stu.sicau.edu.cn (J.-R.Q.); xiangxiangying@stu.sicau.edu.cn (X.-Y.X.); wangjiatong@stu.sicau.edu.cn (J.-T.W.); xuwenxin@stu.sicau.edu.cn (W.-X.X.); chenjia@stu.sicau.edu.cn (J.C.); xiaoyaoximena@stu.sicau.edu.cn (Y.X.); 2 Department of Plant Sciences, University of California Davis, Davis, CA 95616, USA; 3 Crops Pathology and Genetics Research Unit, United States Department of Agriculture, Agricultural Research Service, Davis, CA 95616, USA

**Keywords:** *Myrothamnus flabellifolia*, resurrection plant, phytochrome-interacting factors (PIFs), transcription factor, abiotic stress, abscisic acid (ABA)

## Abstract

Phytochrome-interacting factors (PIFs), a subfamily of basic helix-loop-helix (bHLH) transcription factors (TFs), play critical roles in regulating plant growth and development. The resurrection plant *Myrothamnus flabellifolia* possesses a noteworthy tolerance to desiccation, but no PIFs related to the response to abiotic stress have been functionally studied. In this study, a dehydration-inducible *PIF* gene, *MfPIF1*, was cloned and characterized. Subcellular localization assay revealed that MfPIF1 is localized predominantly in the nucleus. Overexpression of *MfPIF1* in *Arabidopsis* *thaliana* led to enhanced drought and salinity tolerance, which was attributed to higher contents of chlorophyll, proline (Pro), soluble protein, and soluble sugar, activities of antioxidant enzymes as well as lower water loss rate, malondialdehyde (MDA) content, and reactive oxygen species (ROS) accumulation in transgenic lines compared with control plants. Moreover, *MfPIF1* decreased stomatal aperture after drought and abscisic acid (ABA) treatment, and increased expression of both ABA biosynthesis and ABA-responsive genes including *NCED3*, *P5CS*, and *RD29A*. Overall, these results indicated that *MfPIF1* may act as a positive regulator to drought and salinity responses, and therefore could be considered as a potential gene for plant genetic improvement of drought and salinity tolerance.

## 1. Introduction

Various abiotic stress responses are induced by the signal of environmental stresses, for instance, drought, salinity, and high irradiance when plants suffer from adverse circumstances. These responses are controlled by a wide range of sophisticated mechanisms. The interactions of many mechanisms maintain membrane stability, balance osmotic pressure, and reduce damages of active oxygen [1,2], or participate in signal transduction and transcriptional regulation [3], which are partially induced by gibberellin (GA), abscisic acid (ABA), ethylene (ET), jasmonates acid (JA), and salicylic acid (SA) [4,5]. The WRKY, ZFP, bHLH, MYB, NAC, bZIP and DREB transcription factors (TFs) from various families play important roles in the gene regulatory network under unfavorable environments. Overexpressing these stress-related genes in plants has demonstrated an enhanced tolerance to different abiotic stresses [6,7,8].

The basic helix-loop-helix (bHLH) is a large TF superfamily extensively existing in plants, which can be subdivided into 26 subgroups [9]. Phytochrome-interacting factors (PIFs), belonging to the bHLH subgroup 15 of the *A. thaliana* bHLH superfamily, are sensitive to changing light environments, especially to dark circumstances that could disturb the photomorphogenesis of seedlings [10,11]. Hitherto, seven PIFs have been found in *Arabidopsis* including PIF1 (or PIF-like5), PIF3, PIF4, PIF5 (or PIF-like6), PIF6 (or PIF-like2), PIF7, as well as PIF8 [12]. As bHLH transcription factors, every PIF has one highly conserved bHLH domain, containing a basic region and an HLH region followed closely. Binding to specific DNA sequences and promoting protein–protein interactions are realized by the basic region and HLH region, respectively [13,14]. One *N*-terminal active phytochrome B-binding (APB) domain which is highly conserved exists in all PIFs that specifically interact with light-activated phytochrome B (phyB) [15]. Besides, the active phytochrome A-binding (APA) domain which is indispensable for the interaction between phytochrome A (phyA) and PIFs, is found in PIF1 and PIF3 with different critical residues [16,17].

Through the connection of phytohormone signaling networks, members of the PIF family can jointly assist plants in coping with various abiotic stresses. The phytohormone ABA plays an important role during adaptation to drought and salinity stresses like stomatal aperture regulation to maintain the osmotic balance in plant cells [18,19]. A previous study has shown that the deficiency of phyB could enhance water retention capacity and improve drought tolerance in rice by lowering the stomatal density [20]. Furthermore, phyB mutants also enhanced the plant tolerance to drought in *Arabidopsis* mature plants via making the stomata become more sensitive to ABA under water deficiency [21]. The up-to-date research shows that phyA and phyB negatively regulate salinity tolerance of tobacco through ABA-JA synergistic cooperation [22]. Moreover, PIF4 has been reported to be involved in phyB-mediated stomatal ontogeny which is induced by light, but PIF3, PIF5, and PIF6 seem to not be involved [23]. Double overexpression of *OsPIL1* and *DREB1A* improves drought stress tolerance; meanwhile, transcriptome analyses proved that these two TFs work independently [24]. Maize *ZmPIF1* and *ZmPIF3* are positive regulators of drought tolerance via participating in ABA signal networks and controlling stomatal openings to reduce water loss [25,26,27].

PIF1 is the first PIF that regulates seed germination and plays a key role in inhibiting light-dependent seed germination [28]. GA is a plant hormone that positively regulates seed germination, and PIF1 can directly or indirectly inhibit GA signal transduction. Oh et al. showed that PIF1 can inhibit the expression of *RGA* and *GAI* in GA signal transduction through direct activation, which plays an inhibitory role in the GA signaling pathway that promotes seed germination [29]. Meanwhile, PIF1 can inhibit the biosynthesis of GA by activating SOM, thereby inhibiting the normal germination of seeds in dark conditions [30]. In addition, PIF1 can regulate chlorophyll biosynthesis and plastid development in cells [31,32]. Similar to GA, the light signaling pathway is also closely related to the ABA signaling pathway. On the one hand, PIF1 plays an important role in regulating gene expression related to ABA biosynthesis and promoting ABA biosynthesis. On the other hand, PIF1 directly activates the expression of ABA signaling transcription factors ABI3 and ABI5. These TFs not only promote ABA biosynthesis and signal transduction, but also inhibit GA signal transduction and seed germination [33,34]. Besides, PIF1 can also interact with two important regulatory proteins, HFR1 and LEUNIG_HOMOLOG, activate or inhibit the expression of downstream genes between the ABA and GA signaling pathways, and finally affect seed germination [35,36]. This indicates that PIF1 can regulate endogenous ABA biosynthesis and ABA signaling network. In view of the fact that ABA plays an irreplaceable role in the resistance of plants to abiotic stresses, it is inferred that PIF1 is very likely to enhance the abiotic stress tolerance of plants through the ABA transduction pathway. However, the mechanism of PIF1 in drought and salt stresses is far from being understood.

Resurrection plants can keep alive without suffering permanent injury, even if their vegetative organs confine in massive dehydration, and the ability to tolerate the nearly complete desiccation state of their vegetative organs is called desiccation tolerance (DT) [37]. *Myrothamnus flabellifolia* Welw. (Myrothamnaceae), a short shrub from southern Africa, is known as the only wooden resurrection plant for its DT features [38]. To survive in the extremely dry mountain environment, the unique fan leaves of *M. flabellifolius* can fold and roll up tightly when plant tissue is dehydrated, which makes the plants turn quickly into a long-term desiccant state and rehydrate rapidly after contact with water [39,40]. In the meantime, the primary problem faced by photosynthesis under drought stress is that photosynthesis agencies can provide the possibility of generating toxic reactive oxygen species (ROS) [41]. Resurrected plants inhibit the production of ROS by reducing the interaction of light and chlorophyll, and they can also be removed by antioxidants. As a homoiochlorophyllous resurrection plant, *M. flabellifolia* can maintain low-intensity photosynthesis without destroying the photosynthetic mechanism, thereby resisting severe drought stress [42]. Despite a number of physiological and biochemical subjects in *M. flabellifolia* being studied, the molecular mechanisms of extreme tolerance to desiccation and the ability to revitalize still remain unknown. A previous study showed that numerous diverse TFs were involved in desiccation tolerance in *M. flabellifolia* through transcriptome sequencing techniques, among them *MfPIF1,* which is upregulated immediately at the early stage of dehydration [43]. However, its functions involved in dehydration response have not been further investigated. In the present work, we report that the heterologous overexpression of *MfPIF1* enhances both drought and salinity stress tolerance and ABA sensitivity in *Arabidopsis*. Considering all these findings, we propose that *MfPIF1* plays a positive regulatory role in resisting drought and salinity stresses in transgenic plants, which may give a reference to molecular breeding to endow plants with tolerance to abiotic stress.

## 2. Results

### 2.1. Isolation and Characterization of MfPIF1

**Figure 1:**
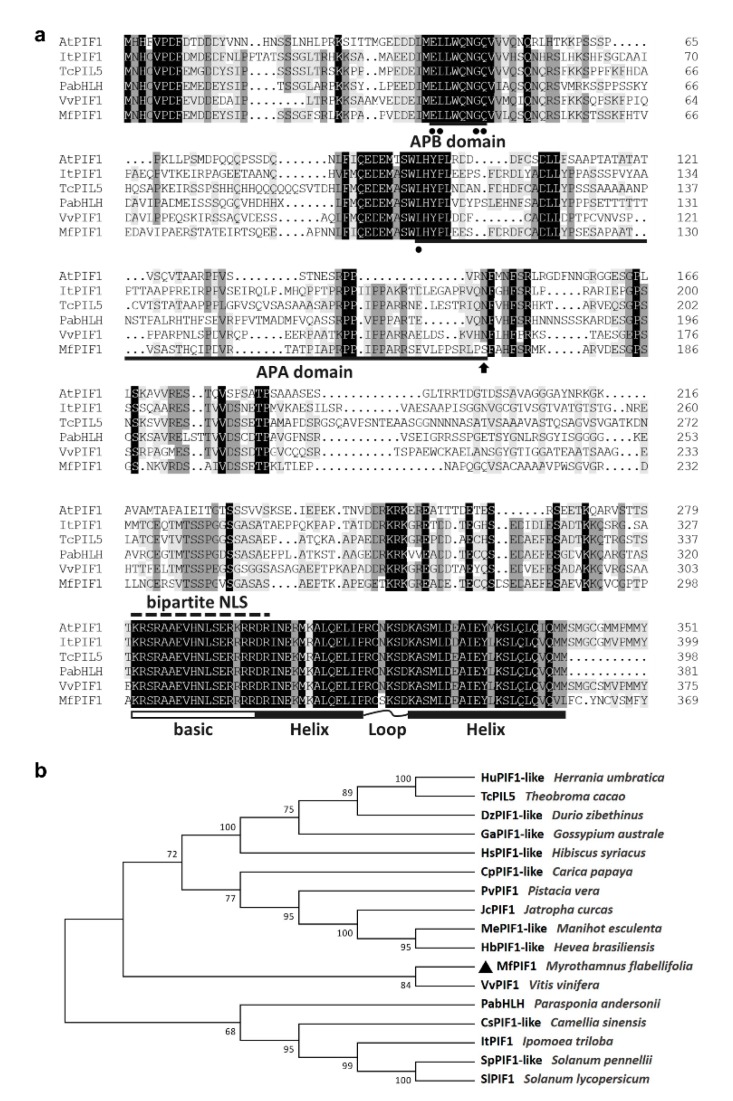
Sequence alignment and phylogenetic analysis of MfPIF1. (**a**) Multiple sequence alignment of MfPIF1 and some highly humongous phytochrome-interacting factors (PIFs). Identical and similar amino acids are shaded in black and gray, respectively. The putative NLS is marked by a dashed line. Active phytochrome B-binding (APB) and active phytochrome A-binding (APA) domains are marked as bold lines, conserved amino acid residues are marked by dots, and the altered amino acid residue at position 169 is indicated by an arrow. The basic region is indicated by a white box, and helix-loop-helix (HLH) conserved domain is labeled with black boxes linked by a curve. (**b**) Phylogenetic tree constructed using neighbor-joining method. MfPIF1 is indicated by a triangle. The GenBank accession numbers are listed in Supplementary Table S1.

The cDNA sequence of *MfPIF1* was cloned from *M. flabellifolia* by PCR amplification. The length of the obtained nucleotide sequence is 1188 bp, which possesses an open reading frame (ORF) encoding 395 amino acids. The protein has a calculated isoelectric point of 5.40 and a predicted molecular mass of 43.76 kDa. A putative bipartite nuclear localization sequence (NLS) “KRSRAAEVHNLSERRRR” at 300 aa was found in the *MfPIF1* protein (Figure 1a). SMART analysis demonstrated that MfPIF1 contains a typical bHLH domain. Moreover, multiple sequence alignment between MfPIF1 and five homologous bHLH proteins indicated that MfPIF1 has an APB domain at its *N*-terminus and a basic region followed by an HLH domain, and these two domains of MfPIF1 had high consistence with those from other plant species. Notably, we also found a putative APA domain in MfPIF1. Unlike the PIF1s of other plants, a conserved amino acid residue at position 169 was changed from asparagine to serine (N to S), which is unique for *M. flabellifolia* among the sequences analyzed (Figure 1a). The subsequent phylogenetic analysis revealed that the MfPIF1 was most humongous to grape VvPIF1 which were grouped to a monophyletic clade (Figure 1b).

### 2.2. MfPIF1 is Localized in the Nucleus of Cells

**Figure 2:**
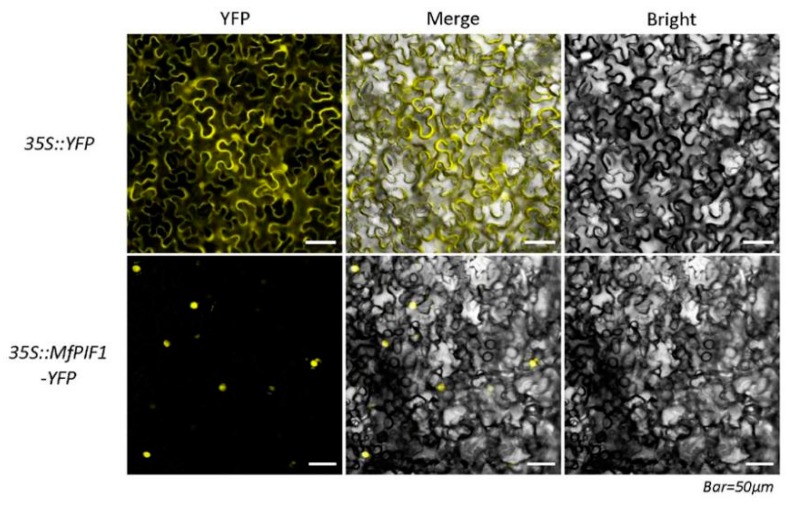
Subcellular localization of MfPIF1 in tobacco epidermis. Fluorescence detection of MfPIF1-YFP fusion protein in tobacco leaf epidermal cells, and tobacco epidermis transformed with 35S::YFP was used as a control (upper lane).

To confirm the above conjecture, we transfected the 35S::MfPIF1-YFP into tobacco leaf epidermal cells for instantaneous expression. Scanning confocal microscopic analysis showed that the fluorescence was detected in the whole cell in 35S::YFP control. On the contrary, the intense yellow fluorescence was nearly exclusively observed in nucleus of 35S::MfPIF1-YFP transformed cell, which proved that MfPIF1 is located in the nucleus (Figure 2).

### 2.3. Overexpressing MfPIF1 Enhanced Tolerance to Drought and Salt

**Figure 3:**
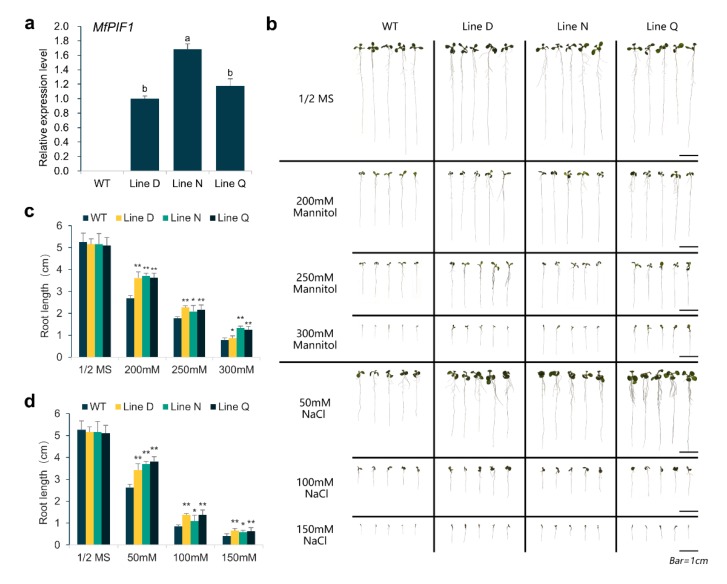
Drought and salinity stress analysis of *MfPIF1* transgenic lines and wild type (WT) at seedling stage. (**a**) Relative expression levels of *MfPIF1* in transgenic plants evaluated by qRT-PCR. Data are presented as mean and SD values of three biological and three technical replicates. Different letters above the columns indicated that the expression levels are significantly different from each other at *p* < 0.05 (LSD multiple comparison test after ANOVA). (**b**) Transgenic and WT seedlings growing on 1/2 MS medium containing mannitol and NaCl for 9 days. (**c**,**d**) Indicated primary root length of nine-day old transgenic and WT seedlings with or without mannitol and NaCl, respectively. Data are presented as mean and SD values of three independent experiments. Asterisks indicate significant difference (* *p*  <  0.05, ** *p*   <  0.01, by Student’s *t*-test) comparing to WT.

To explore the potential function of MfPIF1 in responding to abiotic stress, heterogeneous expression of the *MfPIF1* gene in *Arabidopsis* was performed by constructing a binary vector pGSA1403-*MfPIF1*. T_1_ transgenic *Arabidopsis* lines were acquired from kanamycin resistance screening, and the T_3_ transgenic lines, Line D, Line N, and Line Q, were propagated subsequently. The qRT-PCR results confirmed that expression level of *MfPIF1* could not be detected in wild type (WT), but it was found in all three transgenic lines investigated, with the Line N exhibiting significantly higher expression than the other two lines (Figure 3a). This experiment showed that *MfPIF1* was overexpressed in the measured transgenic *Arabidopsis*, and these three transgenic lines can be selected for subsequent experiments.

In order to investigate if *MfPIF1* is related to drought and salinity stress tolerance, WT and three T_3_ transgenic lines were exposed to the corresponding stress treatments at the seedling stage and adult stage. At seedling stage, no obvious difference between the transgenic and WT plants could be observed when growing in routine conditions (Figure 3b). After mannitol and salt treatments, significantly longer roots were observed in transgenic lines. This difference was more pronounced under treatments with rather lower concentrations of mannitol (200 mM) and NaCl (50 mM) (Figure 3b–d). In a coordinated manner, a clearly large leaf area was also observed in transgenic lines (Figure 3b).

**Figure 4:**
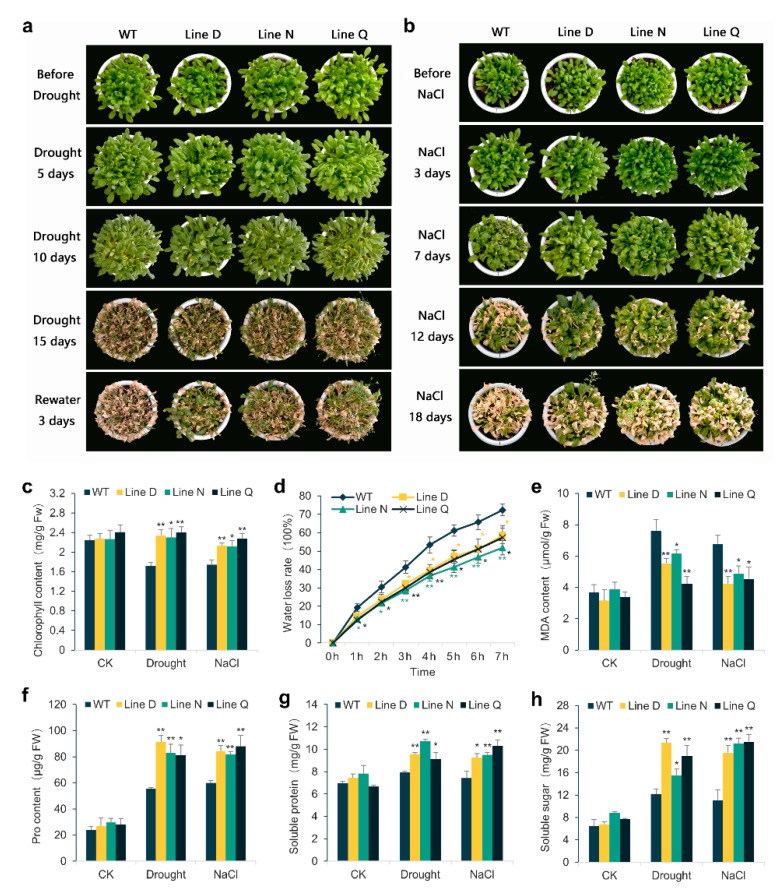
Drought and salinity stress analysis of *MfPIF1* transgenic lines and WT at adult stage. (**a**) and (**b**) show growth status of transgenic and WT plants under drought and salinity conditions. (**c**–**h**) indicate measurements of physiological indexes of *MfPIF1* transgenic lines and WT plants under drought and salinity stresses. (**c**) Changes in chlorophyll content; (**d**) Water loss rates of detached leaves measured at 25 °C room temperature. Malondialdehyde (MDA) (**e**), Proline (Pro) (**f**), Soluble protein (**g**), and Soluble sugar (**h**) contents were measured in transgenic and WT plants. Data are presented as mean and SD values of three independent experiments. Asterisks indicate significant differences (* *p*   <  0.05, ** *p*   <  0.01, by Student’s *t*-test) compared to WT.

For adult stage test, the transgenic and WT plants growing for 4 weeks were cultivated in soil upon drought or 300 mM NaCl treatment to further investigate drought and salt tolerance. No obvious morphological differences were found between the transgenic and WT plants in normal environment, drought for five days, and salt for three days (Figure 4). Ten days after withholding (DAW) watering, slightly withered leaves could be found and the leaf chlorophyll content of *MfPIF1* transgenic lines was 1.34–1.40 times higher than that of WT (Figure 4a,c). At 15 DAW, most of the leaves of the WT plants showed seriously wilting symptoms while some rosette leaves of *MfPIF1* transgenic lines remained fully expanded (Figure 4a). After re-watering for three days, a notable proportion of leaves of transgenic plants recovered rapidly; however, the WT plants were almost dead (Figure 4a).

Negative effects of salinity stress on plant growth became visible after seven days with NaCl treatment (Figure 4b). In the meantime, the leaf chlorophyll content of *MfPIF1* transgenic lines was 1.21–1.31 times higher than that of WT leaves after treatment with salt for 7 days (Figure 4c).When plants were exposed to salinity stress for 12 days, more serious salt injuries were observed on WT plants, whereas transgenic plants were affected to a slight extent of etiolated and wilting symptoms. After 18 days, almost all leaves of WT were withered; however, a considerable portion of leaves of the transgenic plants stayed green and all three lines flowered (Figure 4b).

We measured the water loss rate of rosette leaves at different time points of dehydration. According to Figure 4d, the water loss rates of transgenic plants were remarkably lower than WT in all time points except for 0 h. This result indicated that *MfPIF1* transgenic plants probably had enhanced water-retaining capacity and lost water much more slowly than the WT. Malondialdehyde (MDA), a significant indicator of membrane-lipid peroxidation, can lead to severe damage for the cell membranes. Though the MDA content was elevated in either the WT or transgenic plants under drought and salt treatments, the contents of MDA were significantly lower in all three transgenic lines than WT plants (Figure 4e). Some osmotic adjustment substances can prevent plant cells from dehydrating and improve the tolerance to environmental stress. Thus, we compared contents of several osmolytes, proline, soluble protein and soluble sugar, among three transgenic lines and WT plants with or without drought and salt stresses. As shown in Figure 4f–h, WT and transgenic lines exhibited similar contents of all three osmolytes before treatment. Both stresses increased accumulations of osmolytes in WT and the transgenic lines. Nevertheless, in comparison to WT, all transgenic lines presented remarkably higher contents of proline, as well as soluble protein and soluble sugar.

### 2.4. Effect of MfPIF1 Overexpression on Antioxidant Metabolism in Arabidopsis under Drought and Salinity Stresses

**Figure 5:**
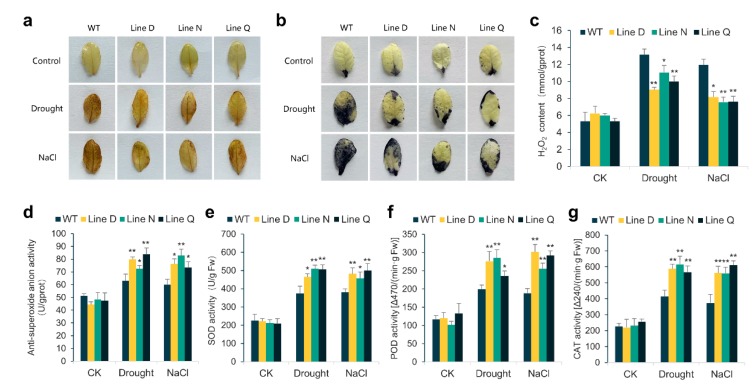
Analysis of reactive oxygen species (ROS) levels and antioxidant enzyme activities in the *MfPIF1* transgenic lines and WT plants under drought and salt treatment. a and b, Histochemical staining with DAB (**a**) and NBT (**b**) were used to detect the accumulation of H_2_O_2_ and O_2_^−^, respectively. (**c**,**d**) showed changes in hydrogen peroxide (H_2_O_2_) content and anti-superoxide anion activity of transgenic and WT plants before and after stress treatments, respectively. (**e**–**g**) indicated activities of superoxide dismutase (SOD), peroxidase (POD), and catalase (CAT) in the leaves of transgenic and WT plants, respectively. Data are presented as mean and SD values of three independent experiments. Asterisks indicates significant difference (* *p*   < 0.05, ** *p*   < 0.01, by Student’s *t*-test) comparing to WT.

As is well known, the cellular oxidative damage aggravates with the level of lipid peroxide rising when plants suffered from abiotic stresses, which is evoked by a large excess of reactive oxidative species (ROS), for instance, hydrogen peroxide (H_2_O_2_) and superoxide anion radical (O_2_^−^), existing in plant cells. Therefore, we used the histochemical staining of DAB and NBT to detect cellular ROS levels under drought and salinity stresses. As shown in Figure 5, the larger leaf areas of WT plants were stained in deeper color than those of transgenic lines (Figure 5a,b), indicating that the transgenic lines underwent less cellular oxidative damage under both stresses. In accordance with these results, less H_2_O_2_ content was detected in three transgenic lines after the drought and salt treatments, which also showed higher anti-superoxide anion activity (Figure 5c,d).

Moreover, we measured antioxidant enzyme activities such as superoxide dismutase (SOD), peroxidase (POD), and catalase (CAT), which are considered as key enzymes in ROS scavenging and are of great relevance to drought and salt tolerance. Consistent with results of ROS level measurement, the activities of SOD, POD, and CAT were significantly enhanced in WT and the transgenic plants upon drought and salt treatments. However, transgenic plants presented apparent stronger activities than those of WT (Figure 5e–g). These results combined proved that overexpression of *MfPIF1* increased capacities of scavenging ROS under stressful conditions, and hence decreased cellular oxidative damage.

### 2.5. MfPIF1 Overexpression Promoted Stomatal Closure Induced by Drought and ABA

**Figure 6:**
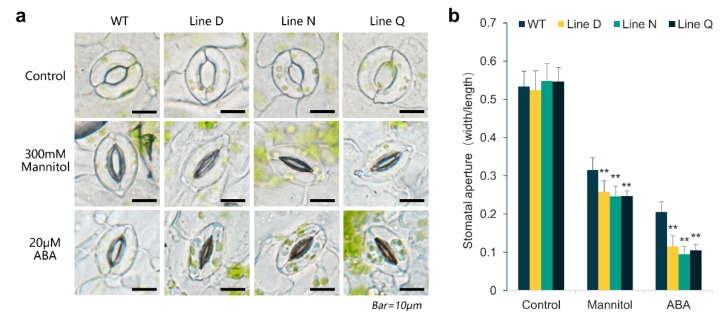
Stomatal aperture of *MfPIF1* transgenic lines and WT plants under 300 mM mannitol and 20 µM ABA treatment. (**a**), Representative images of stomatal aperture in transgenic and WT plants. (**b**), Changes in the stomatal aperture of transgenic and WT plants. The values were calculated as the ratios of stomatal width to length. About 60 stomata were measured for each line. Data are presented as mean and SD values of three independent experiments. Asterisks indicates significant difference (* *p* <  0.05, ** *p* <  0.01, by Student’s *t*-test) comparing to WT.

The response of stomatal movement mediated by ABA plays a central role in transpiration upon drought stress. Hence, we assessed the stomatal closure of leaves under treatments of 300 mM mannitol and 20 µM ABA. Under normal conditions, most of the stomata were opening in all the plants (Figure 6a), and the ratios of stomatal aperture were not significantly different between transgenic and WT plants (Figure 6b). After treatment by mannitol and ABA, the stomatal apertures of three transgenic lines were reduced to 0.25–0.26 and 0.10–0.12, respectively, which were remarkably lower than those of WT plants (0.32 and 0.21) (Figure 6b). These results indicate that the expression of *MfPIF1* promotes stomatal closure in response to mannitol and ABA, which perhaps contributed to the reduced transpiration and decreased water loss rate.

### 2.6. Overexpression of MfPIF1 Up-Regulates Expression Levels of ABA-Responsive Genes

**Figure 7:**
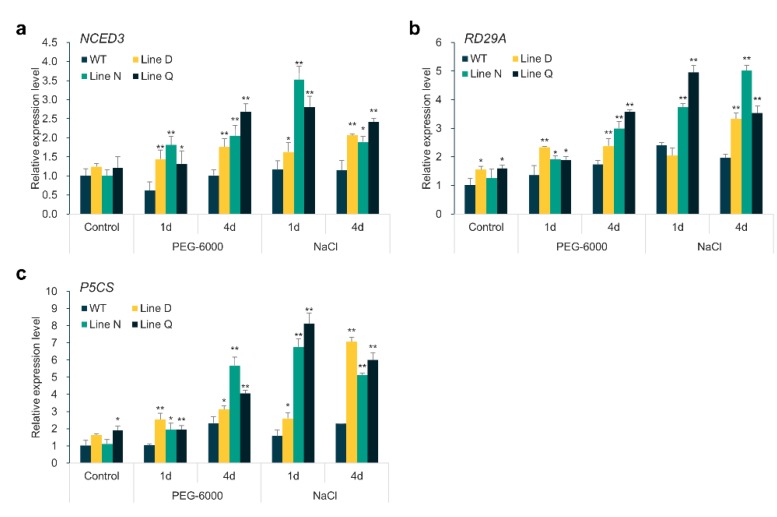
Expression levels of ABA-responsive genes. (**a**–**c**) indicated expression levels of *NCED3*, *RD29A*, and *P5CS* measured by qRT-PCR under normal condition, or treated by 10% PEG-6000 or 300 mM NaCl for 1 d and 4 d, respectively. *AtActin2* was used as an internal reference. Data are presented as mean and SD values of three independent experiments. Asterisks indicates significant difference (* *p*  <  0.05, ** *p*   <  0.01, by Student’s *t*-test) comparing to WT.

To further explore the potential molecular mechanisms for enhanced drought and salinity tolerance in *MfPIF1*-overexpressing lines, we measured the expression quantity of *NCED3*, *P5CS*, and *RD29A* in *Arabidopsis* plants by using qRT-PCR, for one day and four days of artificially simulated drought treatment (10% PEG-6000), or salt treatment (300 mM NaCl). *NCED3* is related to ABA biosynthesis, and all three genes are responsive to ABA, drought, and salinity stresses. Additionally, *P5CS* is involved in proline biosynthesis. As shown in Figure 7, the similar transcription levels of *NCED3* were observed between WT and transgenic *Arabidopsis* under normal conditions. After treatments, the transcription level in WT rose slightly, whereas those in all three transgenic lines increased more rapidly and greatly than the WT, in which Line D responded to salinity stress more slowly than another two transgenic lines (Figure 7a). This might be partially due to the lower gene expression of *MfPIF1* in Line D (Figure 3a). There were higher expression levels of *P5CS* and *RD29A* in transgenic lines than those in WT before drought and salt treatments. Being exposed to drought treatment, the transcription levels of *P5CS* and *RD29A* in *MfPIF1*-overexpressing lines were notably upregulated while slightly increased in WT plants. Under salinity stress, expression levels of *P5CS* and *RD29A* exhibited similar tendency with those observed in *NCED3*. These results suggested that *MfPIF1* positively regulated ABA-responsive gene expression in *Arabidopsis*.

## 3. Discussion

PIFs promote crosstalk among multiple different transcriptional pathways [44]. In recent years, an increasing amount of researches have identified PIFs as key members in the transcriptional pathways underlying abiotic stress, besides the well-known pathways in response to light. The current research reported the characterization of a *PIF* gene *MfPIF1* in *M. flabellifolius*. Sequence analysis suggested that *MfPIF1* contains a conserved bHLH domain, a critical APB domain, and a predicted APA domain (Figure 1a) and showed the highest homology to grape VvPIF1 (Figure 1b). It is localized in the nucleus, suggesting that it may function as a transcription factor to play a basic role in transcription activation or repression.

To investigate whether *MfPIF1* works in the regulation of abiotic stress response, transgenic *Arabidopsis* plants overexpressing *MfPIF1* were generated. According to our results, *MfPIF1* transgenic plants showed better growth status upon drought or salt treatments (Figure 3 and Figure 4), demonstrating that *MfPIF1* acts as a positive regulator to the drought and salinity response in *Arabidopsis*. Plants adapt to adverse environments through morphological, physiological, metabolic, and molecular alterations [45]. Development of a deep root system is a water deficit adaptation strategy that allows the plants to obtain water from the soil to meet the requirement of transpiration [46,47]. In this research, longer primary roots were observed in transgenic seedlings, which may contribute to better water absorption capacity (Figure 3b). Decreased chlorophyll content in drought-stressed or salt-stressed plants is attributed to the inhibition of chlorophyll synthesis [48,49]. MDA as an indicator of lipid peroxidation caused by reactive oxygen species (ROS) could be used for investigating drought and salinity stress tolerance in plants [50]. Hence, the higher chlorophyll content and lower MDA content of *MfPIF1* transgenic plants after stress treatment further confirmed the enhancement of drought and salinity tolerance (Figure 4c,d).

It is well accepted that drought and salinity, commonly presented as osmotic stress, can exert serious injuries in plants. To minimize the damages from osmotic stress, proline, soluble protein, and soluble sugar contents can increase and improve the water holding capacity of leaves by facilitating osmoregulation [51,52,53]. Our studies showed that *MfPIF1* transgenic lines improved the contents of proline, soluble protein, and soluble sugars under drought or salt conditions (Figure 4f–h) which proved that *MfPIF1* may contribute to accumulation of osmolytes to regulate their osmotic potential.

Metabolism processes in plant cells are susceptible to the negative effects of drought and salinity stress conditions. Unbalanced metabolism will lead to oxidation stress in cells by both forcing ROS generation and accelerating ROS accumulation, such as H_2_O_2_ and O_2_^−^, leading to oxidation of cell components, affecting normal metabolism activities and destroying organelle integrity [54,55]. From our results, the WT plants showed fairly higher degree of DAB and NBT staining than *MfPIF1* transgenic lines upon stress treatment (Figure 5a,b). In accordance with these results, the remarkably lower content of H_2_O_2_ and higher anti-superoxide anion activity were found in transgenic lines (Figure 5c,d). Plants have developed efficient ROS scavenging systems by increasing various antioxidation enzyme activities, for instance, SOD, POD, and CAT [56,57]. In our study, all three measured antioxidation enzymes exhibited significantly higher activities in transgenic lines when exposed to drought and salt conditions than those in WT (Figure 5e–g), suggesting that overexpression of *MfPIF1* resulted in a strengthened ROS scavenging system and prevented plants from the severe stress-induced oxidative damage.

Phytohormone ABA plays a complicated role in modulating multiple biochemical processes including seed germination, plant growing and development, along with biotic and abiotic stress responses. Under drought and salt adverse circumstances, plants could promote ABA biosynthesis transferring signals in several complicated defense mechanisms, in which the more representative are stomatal movement and relative gene expression [58,59]. As shown in Figure 4d and Figure 6a,b, transgenic lines presented decreased water loss rates and enhanced stomatal closure induced by drought and exogenous ABA. These results indicated that heterologous expression of *MfPIF1* plants increased efficiency of stomata closure to reduce water loss through transpiration under water-limiting conditions.

There are two main pathways regulating the expression of penetration stress-response genes: one is ABA-dependent, and another is an ABA-independent mechanism; meanwhile, crosstalk also occurs between both pathways [60,61]. It has been reported that the increase of ABA content of *M. flabellifolia* during the dehydration process confirmed that ABA can participate in the defense reaction of resurrection plants [62]. Increasing experiments illustrated that PIFs are involved in drought or salinity stress [24,63], among which several transcription networks in abiotic stress responses are regulated by ABA, and these regulatory networks partially overlap with light signaling [25,26,27]. Our data provided evidence that *MfPIF1* increased sensitivity to stomatal movement induced by ABA, indicating that it may function in stress-response regulation through ABA-dependent pathways. Long-term cell dehydration will cause serious damage to plant cells, which is closely related to the oxidative damage caused by the accumulation of ROS during the dehydration process of *M. flabellifolia*. Especially under strong solar radiation, the ROS homeostasis is extremely susceptible. *M. flabellifolia* retains most of the chlorophyll and maintains the integrity of thylakoid membranes during dehydration, and even if definite photosynthesis is kept, it can still minimize the damage caused by photooxidation and cope with dehydration-induced ROS damage by mobilizing the free radical scavenging system, as well as activating antioxidant enzymes and protective metabolites [64]. In addition, resurrection plants accumulate osmolytes including various low molecular weight proteins, sugars, and compatible solutes, for instance, trehalose and sucrose are considered as important osmoregulators of membrane and cytoplasm [65]. The latest research indicates that homoiochlorophyllous plants *Craterostigma pumilum*, *Selaginella tamariscina*, and *Boea hygrometrica* all show the same morphological and physiological adaptation characteristics during dehydration and hydration [66,67,68,69]. Based on these physiological analysis of the DT adaptation characteristics of resurrection plants, we speculate that if these resurrection plants became adapted to high light intensity environments that are also prone to drought/salinity, these two regulatory networks may become connected by having MfPIF1 upstream or downstream of ABA signaling pathways.

Rate-limiting enzyme gene *NCED3* regulates ABA biosynthesis under stress conditions [70]. In the present research, *NCED3* expressive level was remarkably higher in three transgenic lines after exposure to drought and salinity stress (Figure 7a), suggesting that more endogenous ABA might be synthesized in transgenic plants under abiotic stress. Furthermore, two typical genes responding to ABA-dependent abiotic stress, *P5CS*, and *RD29A* [71,72], are significantly upregulated in transgenic *Arabidopsis* upon drought and salinity stresses, and some transgenic lines have higher expression levels even under normal conditions (Figure 7b,c). As a significant response to abiotic stress, plants can increase proline content to maintain the osmotic balance. *P5CS* plays a distinct role in the control of proline biosynthesis and contributes to proline accumulation during abiotic stress [73,74]. *RD29A* expression can be affected by drought and salinity stresses via ABA-dependent signal transduction pathways, which might be due to the existence of ABRE and DRE motifs in its promoter [75]. Lyall et al. performed gene expression analysis in the monocot resurrection plant *Xerophya humilis* during vegetative desiccation, and speculated that the expression of the ABI3 regulator in leaf tissues is activated by a desiccation-responsive pathway. Based on the above research, we hypothesize that *MfPIF1* may respond to drought stress by directly activating these ABRE-binding factors [76]. Altogether, our results presented evidence that *MfPIF1* enhanced tolerance of *Arabidopsis* to drought and salinity stresses by participating in ABA biosynthesis and ABA-dependent stress-responding pathway.

## 4. Materials and Methods

### 4.1. Plant Materials and Growth Conditions

The *M. flabellifolia*, provided by the Department of Plant Science, University of California, Davis, was grown in the greenhouse of Landscape Architecture Department, Sichuan Agricultural University, Chengdu, Sichuan, China. Experimental plants were grown at a neutrophilous day condition (12 h light/12 h dark) at 22 °C/18 °C, 60% relative air humidity and sufficient light in potter pots.

Wild type (WT) *Arabidopsis* ecotype *Columbia* and transgenic lines were cultivated in a mixed media of soil and vermiculite (1:1, *v*/*v)* in plastic pots under 75% relative humidity and long day (16 h light/8 h dark) treatment at 24 °C/22 °C for 4 weeks before treatments. Seeds of transgenic *Arabidopsis* and WT were sterilized by a 1:1 diluted bleach solution for 5 min, then washed three times using sterilized deionized water. Seeds were subsequently placed on 1/2-strength Murashige and Skoog (MS) medium with 0.7% (*w*/*v*) agar and 2% (*w*/*v*) sucrose and adjusted pH to 5.8-6.0. After being vernalized at 4 °C for two days and growing in an illuminating incubator for about 10 days, young seedlings were transplanted to pots in a growth chamber.

### 4.2. Cloning and Bioinformatic Analysis of MfPIF1

General RNA was isolated from *M. flabellifolia* leaves using Plant Total RNA Isolation Kit (TINAGENE Co., Beijing, China). Synthesis of the first strand cDNA was then prepared with Reverse Transcriptase M-MLV (RNaseH-) (Takara Bio, Dalian, China) with the instruction of kits. The coding DNA sequence (CDS) of *MfPIF1* was amplified by Phanta Max Super-Fidelity DNA Polymerase (Vazyme Biotech Co., Nanjing, China) with a pair of special primers with *Sma*I or *Spe*I restriction site (Supplementary Table S2). Purified PCR products were cloned to a pEasy-T1 Simple vector (TransGen Biotech, Beijing, China) and the construct of the pEasy-T1-MfPIF1 transfer vector plasmid was transformed into strain DH5α *E. coli*, and confirmed by TsingKe Biotech Co., Beijing, China.

The ORF of the nucleic acid sequence was extrapolated based on NCBI ORFfinder (https://www.ncbi.nlm.nih.gov/orffinder/). Isoelectric point (pI) and molecular weight of the MfPIF1 protein were determined by ExPASy (https://web.expasy.org/compute_pi/). Conserved domains of the deduced protein sequence were analyzed with SMART (http://smart.embl-heidelberg.de/). Comparison of amino acid sequences of MfPIF1 with other close homologs from different species was done using NCBI software BLASTP (https://blast.ncbi.nlm.nih.gov/Blast.cgi). Multiple alignments between MfPIF1 and its homologous proteins were performed using DNAMAN v. 9.0 software. A phylogenetic tree was obtained through software MEGA v. 7.0 [77] based on the neighbor-joining approach, and the bootstrap test was replicated 1000 times. The prediction of protein secondary structure was studied with Jpred 4 (http://www.compbio.dundee.ac.uk/jpred/index.html).

### 4.3. Subcellular Localization of MfPIF1

The full-length ORF without the termination codons of *MfPIF1* was amplified using primers with homologous arm sequences (Supplementary Table S2). The confirmed product was double-digested using *Hind*III and *BamH*I, followed by inserting into the pHB-YFP vector to construct a fusion expression vector MfPIF1-YFP driven by a CaMV (Cauliflower Mosaic virus) 35S promoter. Sequence-verified constructs 35S::MfPIF1-YFP and 35S::YFP were introduced into the *Agrobacterium tumefaciens* (*GV3101*) cells using the freezing-thawing method. Leaves of wild-type tobacco growing to 4 weeks old (*Nicotiana benthamiana*) were injected with suspensions including either the fusion construct or the control vector (YFP alone). All the transformed tobaccos were cultured at 22 °C in the dark for 16 h and then cultured for two days before observing the expressed of YFP by a laser confocal scanning microscope (Nikon, Tokyo, Japan).

### 4.4. Vector Construction and Generation of Transgenic Lines

To produce *35S::MfPIF1* lines, the encoding region of *MfPIF1* was PCR-amplified with primers containing either a *Sma*I or *Spe*I restriction site (Supplementary Table S2), and the amplicon was linked into the same enzyme recognition sites of the plant binary vector pGSA-1403, resulting in the construct pGSA1403-*MfPIF1* under the control of the CaMV 35S promoter. After that, the recombinant plasmid *35S::pGSA1403-MfPIF1* was transformed into the *A. tumefaciens* LBA4404, and *Arabidopsis* plants were transformed using the floral-dip transformation method [78]. The first generation T_0_ seeds of *MfPIF1* transgenic plants were collected and selected by 1/2 MS culture medium containing kanamycin (50 µg/mL). Kanamycin-resistance seedlings were transplanted to pots with soil for the following studies. Positive transgenic plants were detected with PCR with gene-specific primers as described above. Three homozygous T_3_ positive lines were selected for further tolerance studies and other experiments.

### 4.5. Expression Analysis of MfPIF1 and ABA-Responsive Genes

Leaves from transgenic and WT seedlings growing to 4 weeks old, incubated under normal conditions, for one day and four days under simulating drought stress with PEG-6000 (10%) or salinity stress with 300 mM NaCl were used for expression analysis. General RNA from different lines was extracted with Plant RNA Kit (Omega Bio-tek, Norcross, GA, United States), which was also purified with RNase-Free DNase I (Omega Bio-tek) and reverse transcribed into cDNA by use of Uscript II (Innovagene biotech, Hunan, China).

The qRT-PCR amplification was performed in 25 µL reaction mixture (innovagene biotech) including 12.5 µL 2 × Taq SYBR Green qPCR Mix, 0.5 µL of 10 µM of each primer, 4 µL 5-fold diluted cDNA, and 7.5 µL of Nuclease-free H_2_O, which was performed using the real-time PCR instrument CFX Connect (Bio-Rad, Hercules, CA, USA). Amplification conditions were devised of 94 °C for 3 min, 42 cycles of degeneration at 94 °C for 8 s, annealing/extension at 60 °C for 60 s. The relative expression quantity of the target gene was evaluated based on the method of 2^−ΔΔCT^ [79]. Finally, the results were normalized by an internal reference gene *AtActin2* for quantitative analysis of relative genes. Each RT-qPCR experiment was reproduced at least three times. The gene-specific amplification primers were listed in Supplementary Table S2.

### 4.6. Assays of Drought and Salinity Stress Tolerance

For seedling stage stress assays, sterilized seeds of transgenic and WT plants were placed on 1/2 MS solid medium containing varying concentrations of mannitol (0, 200, 250 or 300 mM) and NaCl (0, 50, 100 or 150 mM). Culture dishes were settled vertically in the light incubator in a cycle for 16 h light (24 °C) and 8 h dark (22 °C). After growing 9 days, the taproot length of each line (15 seedlings every petri dish, and three dishes in total) was measured. Each experiment was performed in three replicates.

In order to explore the tolerance for mature plants to drought and salinity stress, the same amount of soil was added into pots in same size and well watered. Approximately 50 seeds for WT plants and three T_3_ transgenic lines were vernalized at 4 °C for two days and evenly sown into pots with regular cultivation (watering same amount of water regularly). Four weeks later, each pot was treated with drought or salt. In the case of drought treatment, ample water was irrigated in the tray before drought stress treatment to make the soil water content reach saturated water capacity, and after the seedlings recovered for 24 h, plants were subjected to drought stress through stopping watering over 15 days and then rewatered. For salt treatment, plants were treated by a salt solution with 300 mM NaCl twice at 3-day intervals. During the stress treatment, the real salt concentration of soil increased gradually. All pots were photographed every two or three days, and three days after re-watering, and also before the drought or salt stress. Samples of 4-week-old plants for physiological index measurements under stress conditions were obtained through drying treatment over 10 days and salt (300 mM NaCl) over 7 days. These assessments were conducted in triplicate.

### 4.7. Estimation of Water Loss Rate

To figure out the change of water loss rate, 0.5 g rosette leaves from WT plants and three T_3_ transgenic lines grown for four weeks were excised and promptly weighed. The leaves in the same status from each line were then laid on an experiment table and weighed at setting time points (0, 1, 2, 3, 4, 5, 6, and 7 h), with three replicates for each line. Water loss percentage can be obtained according to the fresh weights of isolated leaves before dehydration.

### 4.8. Physiological Measurements

Chlorophyll was extracted from leaf tissue in 95% ethanol as previously described [80]. Proline was measured following the modified method of acidic ninhydrin reaction as stated earlier [81]. The soluble protein and soluble sugar content were determined using TP quantitative assay kit (Nanjing Jiancheng, Nanjing, China) and the plant soluble sugar content test kit (Nanjing Jiancheng), respectively, following the user manual instructions. The accumulation of hydrogen peroxide (H_2_O_2_) and superoxide anion radical (O_2_^−^) in leaves were visualized by histochemical staining with 3,3′-diaminobenzidine (DAB) and nitroblue tetrazolium (NBT) separately [82]. Leaves were then decolorized in 95% ethanol before recorded by photograph. The H_2_O_2_ level and anti-superoxide anion activity (an indicator of O_2_^−^ content) were analyzed by hydrogen peroxide assay kit (Nanjing Jiancheng) and inhibition and produce superoxide anion assay kit (Nanjing Jiancheng), respectively, according to the operating instructions. The enzyme liquid was extracted for combined determination of superoxide dismutase (SOD), peroxidase (POD), and catalase (CAT) activities as well as malondialdehyde (MDA) content. Detailed descriptions of these assays were elaborated by Zheng et al. [83] and Du and Bramlage [84]. Three replicates were executed for these experiments.

### 4.9. Analysis of Stomatal Aperture Responsive to Drought and ABA Treatment

To measure stomatal movement induced by drought and ABA, rosette leaves of WT plants and three T_3_ transgenic lines growing for 4 weeks were floated on a solution to induce stomatal opening (50 mM KCl, 0.1 mM CaCl_2_, and 10 mM MES, pH 6.15) and placed under light for 2.5 h. Then, we transferred these leaves into the opening solution with 0, and 300 mM mannitol, or 20  µM ABA and they were incubated in a growth chamber for a further 2 h. Stomata on the leaf lower epidermal layers were immediately observed and photographed by means of an optical microscopy (DP80, Olympus, Japan), and the stomatal aperture (width to length ratio) from 60 stomata of each line was measured. All experiments were repeated in triplicate.

### 4.10. Statistical Analysis

Data from this study were analyzed by Student’s *t*-test in SPSS v. 23.0. The measured values were expressed as the mean ± standard deviation (SD) of three replicates, and differences were viewed as to be significant at * *p* < 0.05 and ** *p* <  0.01.

## Supplementary Materials

Supplementary materials can be found at https://www.mdpi.com/1422-0067/21/8/3011/s1. Table S1: The GenBank accession numbers of MfPIF1 and some highly humongous PIFs used to construct phylogenetic tree in Figure 1; Table S2: List of primers used in this study.

## Abbreviations

PIFs	phytochrome-interacting factors
bHLH	basic helix-loop-helix
TFs	transcription factors
DT	desiccation tolerance
APB	active phytochrome B-binding
APA	active phytochrome A-binding
ORF	open reading frame
NLS	nuclear localization signal
WT	wild type
P5CS	Δ-1-pyrroline-5-carboxylate synthetase
