# Supplementary material for: MfPIF1 of Resurrection Plant Myrothamnus flabellifolia Plays a Positive Regulatory Role in Responding to Drought and Salinity Stresses in Arabidopsis
Source: Int J Mol Sci. 2020 Apr 24;21(8):3011. doi: 10.3390/ijms21083011 (PMC7215370; doi:10.3390/ijms21083011)
Supplement: Supplementary file 1 [file ijms-21-03011-s001.pdf]

International Journal of Molecular Sciences

MDPI

Supplementary

MfPIF1 of Resurrection Plant *Myrothamnus flabellifolia* Plays a Positive Regulatory Role in Responding to Drought and Salinity Stresses in *Arabidopsis*

Jia-Rui Qiu [1], Xiang-Ying Xiang [1], Jia-Tong Wang [1], Wen-Xin Xu [1], Jia Chen [1], Yao Xiao [1], and Cai-Zhong Jiang [2], Zhuo Huang [1,]*

[1] College of Landscape Architecture, Sichuan Agricultural University, Wenjiang, Sichuan, 611130, China

[2] Department of Plant Sciences, University of California Davis, Davis, CA, 95616, USA

* Correspondence: huangzhuo@sicau.edu.cn

Table S1. The GenBank accession numbers of some high humongous PIFs used to construct phylogenetic tree in **Figure 1**.

Genes	Accession numbers
DzPIF1-like	XP_022771394.1
CsPIF1-like	XP_028065178.1
ItPIF1	XP_031122679.1
PvPIF1	XP_031278282.1
GaPIF1-like	KAA3458677.1
HsPIF1-like	KAE8685328.1
HuPIF1-like	XP_021275187.1
TcPIF3-like5	EOX94040.1
HbPIF1-like	XP_021275187.1
PabHLH	PON51411.1
VvPIF1	RVW79758.1
SpPIF1-like	XP_027775414.1
JcPIF1	XP_012085172.1
CmPIF1-like	XP_022953168.1
PaPIF1-like	XP_028758239.1

International Journal of *Molecular Sciences*

MDPI

Table S2. List of primers used in this study.

Usage	Primer names	Primer sequences (5' - 3')
Clone	pGSA1403-MfPIF1-F	TCCCCCGGGATGAATCACTGCGTTCCCGA
	pGSA1403-MfPIF1-R	GACTAGTTTAAAATTTCTCATACCTGTG
Subcellular location	pHB-MfPIF1-YFP-F	*ACCAGTCTCTCTCTC*AAGCTTATGAATCACTGCGTTCCCGA
	pHB-MfPIF1-YFP-R	*GCTCACCATACTAGT*GGATCCAAATTTCTCATACCTGTG
qRT-PCR	AtActin2-F	GGAAGGATCTGTACGGTAAC
	AtActin2-R	TGTGAACGATTCCTGGACCT
	MfPIF1-F	AATTCGGCATCCTCACTGTC
	MfPIF1-R	TGCTGAACTGTGAGCGTTCT
	AtNCED3-F	CGAGCCGTGGCCTAAAGTCT
	AtNCED3-R	GCTCCGATGAATGTACCGTGAA
	AtP5CS-F	GGTGGACCAAGGGCAAGTAAGATA
	AtP5CS-R	TCGGAAACCATCTGAGAATCTTGT
	AtRD29A-F	GATAACGTTGGAGGAAGAGTCGG
	AtRD29A-R	TCCTGATTCACCTGGAAATTTCG

[1] The restriction sites are under-lined and the homologous arm sequences are italicized. F and R represent the forward and reverse primers from 5' end to 3' end.
